# Fluorescence quenching based detection of nitroaromatics using luminescent triphenylamine carboxylic acids

**DOI:** 10.1038/s41598-021-97832-0

**Published:** 2021-09-29

**Authors:** Aamnayee Mishra, R. Dheepika, P. A. Parvathy, P. M. Imran, N. S. P. Bhuvanesh, S. Nagarajan

**Affiliations:** 1 Department of Chemistry, Central University of Tamil Nadu, Thiruvarur, 610 005 India; 2 Department of Chemistry, Islamiah College, Vaniyambadi, 635 752 India; 3 Department of Chemistry, Texas A&M University, College Station, TX 77842 USA

**Keywords:** Materials chemistry, Organic chemistry, Photochemistry

## Abstract

Detection of nitroaromatics employing greener techniques has been one of the most active research fields in chemistry. A series of triphenylamine (TPA) functionalized carboxylic acids were synthesized and characterized using various spectroscopic techniques including single-crystal X-ray diffraction analysis. The interaction of carboxylic acid-decorated TPAs with nitroaromatic compounds was photophysically explored using absorption and emission spectroscopy. Stern–Volmer plot accounts for the appreciable quenching constant of the TPA-acids. Density functional theory calculations were carried out to study the new compounds' frontier molecular orbital energy levels and the possible interactions with picrate anion and revealed an unusual charge transfer interaction between acids and picrate anion. The contact mode detection shows the TPA-acids can be used as dip-strip sensors for picric acid detection.

## Introduction

The easy detection of the toxic and very harmful nitroaromatics for health, environmental and national security reasons has been studied widely by scientists in the last few decades^1–3^. Nitroaromatic compounds (NAC) are potent explosives and also used in various industries such as pharmaceuticals, dye, and other chemical laboratories^4,5^, often cause fatal injuries all around the Globe. Many of the NAC are recognized to be mutagenic or carcinogenic^6^. Hence, detecting NAC with high sensitivity is exceedingly anticipated for health, environmental, and security reasons^7–9^. In earlier days, detecting these hazardous materials involved sophisticated methods^10–14^ and instruments such as GC, HPLC, polarography that are not suitable for real-time applications. Therefore, researchers have been diverted to develop uncomplicated new analytical methods to detect NAC economically in the last two decades^15–17^.

The technique of fluorescence sensing is a selective, sensitive and convenient method to detect various metals or harmful chemicals. Hence much attention has been drawn to designing and synthesizing efficient small organic fluorophores for the detection of nitroaromatics like picric acid (PA)^15,18–20^. Several organic fluorophores have been investigated to detect NAC by fluorescence quenching. Various electron-rich molecules like benzo[k]fluoranthene^21^, anthracene-functionalized tris-imidazolium salt^22^ have been reported to sense PA selectively. In addition, vapor-phase detection of PA has been successfully acquired with PFMI-NP a conjugated polymer nanoparticle^23^. Recently tertiary amine-functionalized imidazole-based boron complexes have been reported for selective detection of PA^24^. Solid-state sensing also provides promising results on the high vapor pressure of nitrobenzene leading to a decrease in fluorescence quenching^25^. It also showcases the use of paper strips for contact mode detection of PA.

Triphenylamine (TPA) is a suitable class of fluorophores widely used because of their unique geometry and electron-donating property, which provides a suitable optical platform to be modulated for detecting various metal ions as well as harmful compounds^18,20,26^. TPA has always been used as a crucial molecule taking part in ICT^27^ making it the widely used molecule for optoelectronic applications^28,29^. Palas et al.^30^ reported that strong donor ability of the molecule with extensive π—delocalization may lead to better interaction between electron-deficient PA and the molecule. Duraimurugan et al.^20^ reported the change in colour of solution due to charge transfer complexation with picrate anion. The work of Chowdhury and Mukherjee on the synthesis of acid groups substituted triarylamines derivatives is worthy to mention here^26^. They established the existence of a charge-transfer band which was based on the UV–Vis studies. Solvents such as *N,N*-dimethylformamide (DMF), *N,N*-Dimethylacetamide (DMA), dimethyl sulfoxide (DMSO), PA undergoes deprotonation to form picrate anion, which in turn participated in ground-state charge-transfer complex formation.

Herein we report the design and synthesis of new TPA molecules decorated with the carboxylic acid group(s) for the detection of NACs through fluorescence sensing. Often fluorescent pH probes pertaining to the availability of H^+^ are of greater significance due to various advantages such as non-destructive character, high sensitivity, and specificity^22,31^. It is essential to design the fluorescent sensors which would easily display fluorescence enhancements, high sensitivity, and selectivity towards the analyte molecule, which inspired us to use the potential –COOH functional groups as they can be easily converted to other activating receptors by forming a PET system^21,32^. Due to the delocalized ${\uppi }^{*}$ excited state, compound’s electron density is modulated, which facilitates interaction with electron-deficient nitroaromatic analytes. The interactions are scrutinized by absorption and emission spectroscopic techniques. Multiple fluorescent units amplify the fluorescence quenching through energy and electron transfer and as a result, the interaction studies become efficient. Solution and contact mode detections are also investigated as the cost-effective methodology for NAC detection. This investigation can provide a pathway for selective detection of NAC by the fluorescence quenching method.

## Experimental section

### Materials

All the materials (triphenylamine, 4-aminobenzonitrile, 4-flurobenzonitrile, phosphorous(V) oxychloride (POCl_3_), potassium permanganate(VII) (KMnO_4_), dimethyl sulfoxide (DMSO), potassium hydroxide (KOH), cesium fluoride (CsF), *N,N*-dimethylformamide (DMF), acetone (CH_3_COCH_3_), ethanol (EtOH)) were used as received from the commercial sources. ACS grade solvents were used without further purification. Column chromatography was carried out with slurry-packed activated silica gel (100–200 mesh).^1^H and ^13^C NMR and was recorded using Bruker 400 MHz instrument in CDCl_3_ and d_6_ DMSO.

UV–Vis absorption spectra were recorded using a Jasco V-670 spectrophotometer. Perkin Elmer LS 55 spectrofluorimeter was used to obtain the emission spectra. The electrospray ionization mass (ESI–MS) spectra were recorded using THERMO Scientific Exactiveplus UPLC mass spectrometer. Single-crystal x-ray diffraction studies data were collected on a BRUKER APEX2 X˗ray (three˗circle) diffractometer with Mo˗kɑ λ = 0.70173 Ǻ radiation. Microwave-assisted synthesis was performed using a CEM microwave synthesizer. For density functional theory (DFT) studies, the molecules were optimized at the 6–31 D basis level of DFT using Gaussian. The frontier molecular orbital values i.e. highest occupied molecular orbital and lowest unoccupied molecular orbital (HOMO and LUMO) were also extracted.

### Synthesis

#### General procedure for the synthesis of compounds 2–4

TPA acids were synthesized as per the reported procedures^33,34^. In brief, compounds **2** and **3** were synthesized by a two-step synthetic sequence: commercially available triphenylamine (**1**) was subjected to Vilsmeier Haack formylation followed by oxidation using KMnO_4_ in acetone: water (4:1). Compound **4** was prepared by hydrolysis of 4,4’,4″-tricyanotriphenylamine using aqueous KOH via microwave-assisted synthesis (Scheme 2).

##### 4-(Diphenylamino)benzoic acid (Compound 2)

To a stirred solution of TPA mono aldehyde (1.5 g, 5.49 mmol) in 50 mL acetone–water (4:1), KMnO_4_ (4 g, 25.16 mmol) was added portion-wise at 60 °C for 1 h. The reaction was stirred under reflux conditions for 4 h. The acetone was removed under vacuum and water (25 mL) was added and filtered. The filtrate was acidified with HCl to give a white precipitate. The precipitate was filtered and washed with water several times and dried under a vacuum to give compound 2. Pale yellow solid. Yield: 80%. ^1^H NMR (400 MHz, d_6_ DMSO, δ in ppm): 7.80–7.78 (d, J = 8.0 Hz, 2H); 7.38 (t, J = 7.2 Hz, 4H); 7.17–7.12 (m, 6H); 6.88–6.86 (d, J = 8.4 Hz, 2H); 3.48 (s, 1H). ^13^C NMR (100 MHz, DMSO) δ 167.5, 151.8, 146.5, 131.3, 130.3, 126.2, 125.2, 122.8, 119.4. HRMS (ESI) calculated for C_19_H_15_NO_2_ [M + H]^+^: 290.1136; found, 290.1183.

##### 4,4’-(Phenylazanediyl)dibenzoic acid (Compound 3)

4,4’-Diformyltriphenylamine (0.2 g, 0.66 mmol) was dissolved in a mixture of 15 mL acetone: water (4:1), and the solution was heated. KMnO_4_ (0.5 g, 3.3 mmol) was added portion-wise to the mixture for one hour and the reaction mixture was warmed to 60 °C and stirred for 5 h. After removal of acetone, water (10 ml) was added to the residue and the mixture was filtrated and was acidified by slow addition of concentrated HCl. The white precipitate was filtered and dried under a vacuum to afford the product. Crude was recrystallized from DCM and methanol. Yellow small square-shaped crystals formed. Yield: 66%.^1^H NMR (400 MHz, d_6_ DMSO, δ in ppm): 12.72 (s, 2H); 7.88–7.86 (d, J = 8.4 Hz, 4H), 7.43 (t, J = 5.9 Hz, 2H); 7.26–7.152 (m, 2H); 7.07–7.05 (d, J = 22.4 Hz, 4H). ^13^C NMR (100 MHz, DMSO) δ 167.2, 150.8, 146.0, 131.4, 130.6, 126.9, 126.0, 125.0, 122.5. HRMS (ESI) calculated for**:** C_20_H_15_NO_4_ [M]^+^:333.3374; found: 333.1008.

##### 4,4′ 4″-nitrilotribenzoic acid (Compound 4)

A mixture of 4-aminobenzonitrile (0.05 g, 0.423 mmol), 4-flurobenzonitrile (0.11 g, 0.93 mmol), CsF (0.25 g, 1.69 mmol), and 2 mL dry DMF were heated at 140 °C for 20 min under microwave conditions (Power: 155 W, pressure: 250 psi, pre-stirring time: 1 min). Progress of the reaction was monitored by TLC. After the completion, the reaction mixture was poured into ice-cold water. The solid product was filtered and washed with plenty of water. The product was dried under a vacuum. The crude product was dissolved in EtOH: KOH (3:1) and heated at 110 °C for 20 min under microwave conditions (Power: 155 W, pressure: 250 psi, pre-stirring time: 1 min). Completion of the reaction was monitored by TLC. After cooling down to room temperature the mixture was acidified with 3 N HCl to pH 3 to get a white precipitate. Finally, the precipitate was filtered and washed with plenty of water, dried in an oven to obtain the desired product. Pale yellow solid. Yield: 80%. ^1^H NMR (400 MHz, d_6_ DMSO, δ in ppm): 12.81 (s, 3H); 7.92–7.90 (d, J = 7.2 Hz, 6H); 7.15–7.13 (d, J = 7.2 Hz, 6H). ^13^C NMR (100 MHz, DMSO) δ 167.1, 150.2, 131.6, 126.3, 124.1. HRMS (ESI) calculated for: C_21_H_15_NO_6_ [M-H]^−^:376.0821; found 376.0828.

### Preparation of solutions

Stock solutions (10^−3^ M) were prepared by weighing the calculated amount of the compounds and dissolving in the calculated volume (10 mL) of DMSO solvent. Further dilution was made for desired concentration for photophysical studies. Similarly, the stock solutions (10^−3^ M) of NAC were made by dissolving the calculated amount of appropriate compound in DMSO solvent. Solutions were diluted further for photophysical studies.

### Photophysical studies

For UV–Vis absorption, the TPA compounds at 10^−5^ M concentration were titrated against nitroaromatics (10^−4^ M), and the corresponding responses were recorded. 100 µL of particular NAC was successively added to 2 ml of the compound for photophysical studies. For Fluorescence titration, dilute solutions of the compounds **1–4** (10^−7^ M) were titrated with nitroaromatics (10^−5^ M), particularly for PA response was recorded for the compounds **1–4** in 10^−6^ M.

## Results and discussions

**Scheme 1:**
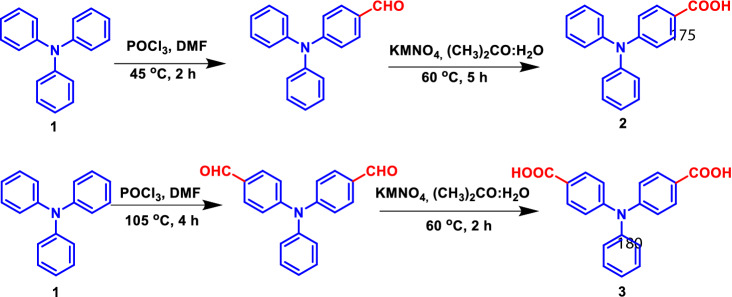
Synthesis of compounds **2** and **3.**

**Figure 1:**
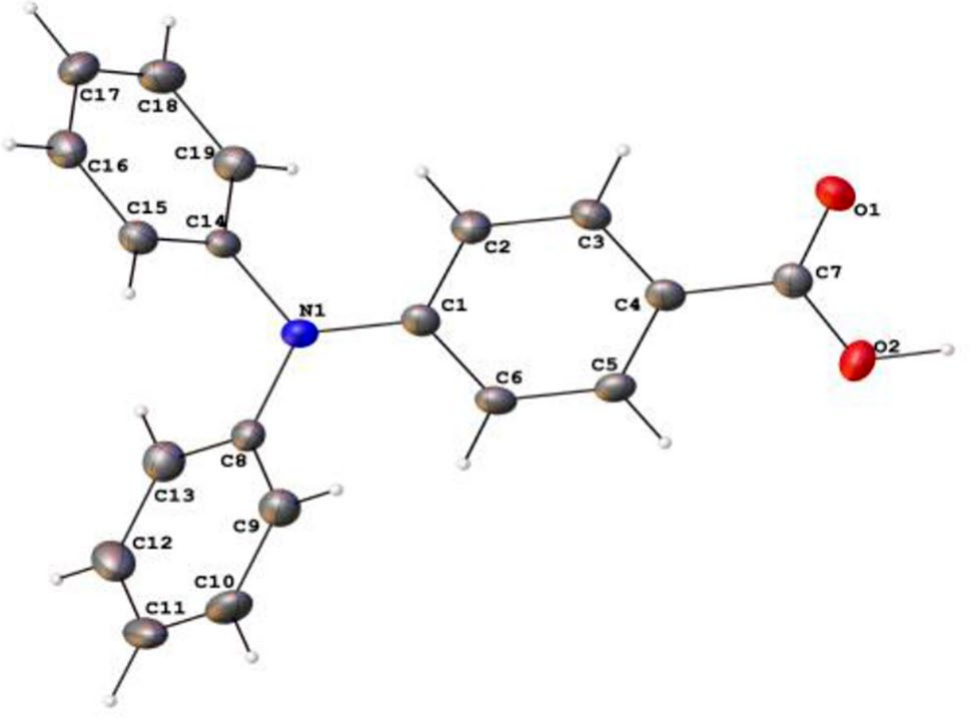
The thermal ellipsoidal plot of compound 2 (TPA monoacid). The unit cell dimensions are a = 9.1921(6) Åα= 90°.b = 9.6185(7)Å β = 90.068(2)°.c = 17.1694(11) Å γ = 90°. The crystal system is monoclinic with a volume of 1518.02(18) Å^3^.

**Scheme 2:**
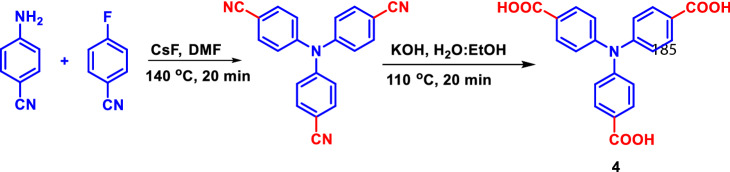
Microwave-assisted synthesis of compound **4.**

TPA can be used as a three-armed fluorescent probe^35^, due to its high fluorescent nature and solubility in a wide range of solvents. The substitution will alter its photophysical properties. Recent reports suggest that TPA molecules are used as sensors to detect harmful chemicals and biologically important metal ions^18,20,26,30^. Hence, we designed and synthesized mono, di, and tri TPA carboxylic acids with modulated electron density for sensing applications. In addition, deprotonation of carboxylic acids will also lead to some interesting studies. The synthetic pathway is given in Scheme 1 and 2. Quenching of emission intensity of an electron-rich fluorophore by electron-poor nitroaromatics is associated with an intermolecular photoinduced donor–acceptor electron transfer mechanism. All the synthesized compounds are well characterized by NMR, spectroscopic techniques including single-crystal XRD (Fig. 1).

### Optical properties

The absorption spectra of the compounds **1**–**4** in DMSO show a single absorption peak in the range of 300–348 nm, corresponding to π-π* transition of the conjugated TPA core. For compound **3** in addition, a shoulder peak is also obtained at 317 nm which might be due to the intramolecular charge transfer (ICT).

**Figure 2:**
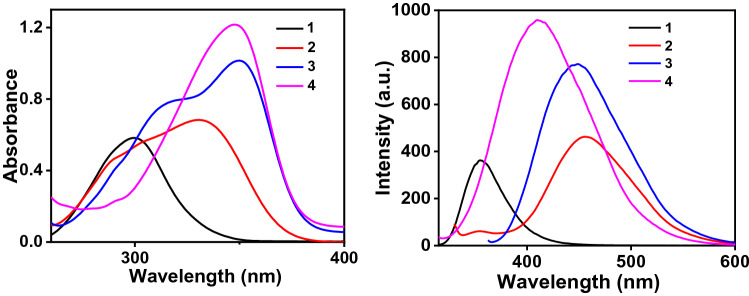
(**a**) UV–Vis absorption and (**b**) emission spectra of compounds 1–4.

The absorption and emission spectra are shown in Fig. 2a,b, respectively. When the medium is polar high charge separation leads to a change in dipole moment and instigates the molecule to attain a different geometry in the excited state. The excited state transitions involved (in vibrational levels of *S*_1_ and *S*_0_) are different than that of the absorption in the case of compounds **2** and **3** (TPA monoacid and di acid respectively) resulting in the breakdown of mirror-image symmetry^36–38^.

The area under the peak is increasing with the increase of the number of carboxylic acid groups, concluding the fact that an increase in the number of acid groups increases fluorescence intensity, binding capacity thereby using the effect of the autochrome –COOH. Compound **2** shows the most significant Stoke’s shift of 126.8 nm indicating a large change in the dipole moment of the molecule after excitation relevant to Table 1 confirming its value to be 7.15D. Polar solvent DMSO activates the probable intermolecular charge transfer from one part (donor) of the molecule to the other (acceptor) in the excited state. While **4** (TPA tri acid) shows the least vale of 64.0 nm among the three acids as well as compound **4** (TPA tri acid) has the highest absorption coefficient of 12.1 × 10^4^ L mol^−1^ cm^−1^ making it an interesting molecule for fluorescence studies.

**Table 1:** Photophysical properties of compounds **1**–**4.**

C. no	λ_Abs_ (nm)	λ_Em_ (nm)	Stokes’ shift (nm)	Absorption co-efficient (ε, 10^4^ L mol^−1^ cm^−1^)
1	300	355.5	55.5	5.8
2	331	457.8	126.8	6.8
3	350	445.9	95.9	10.1
4	348	412.0	64.0	12.1

### Interaction with nitroaromatics

**Figure 3:**
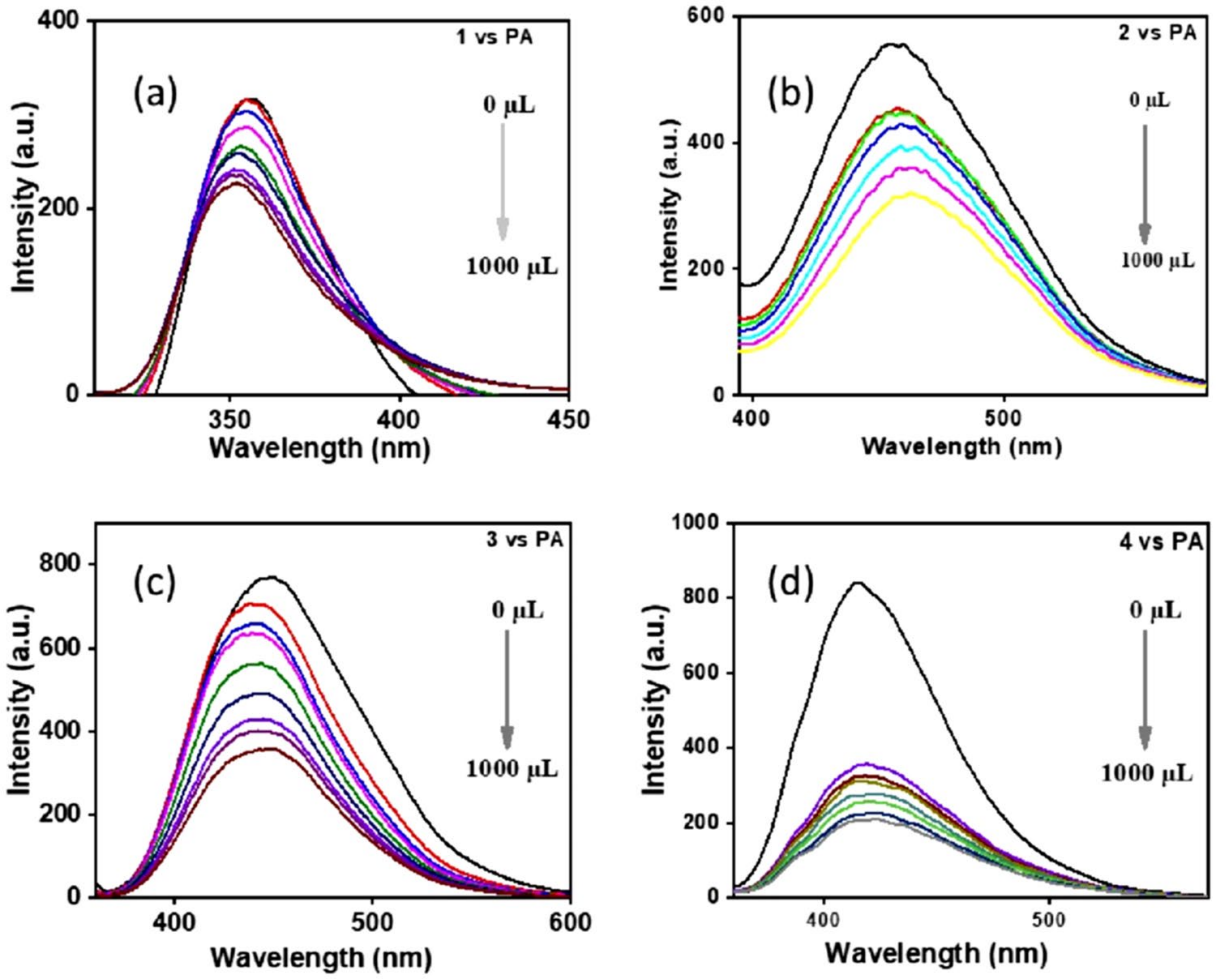
Fluorescence spectra of compounds **1**–**4** in the presence of picric acid (PA). The concentration of picric acid in DMSO is in the order of 0 M, 4.76 $\times$ 10^−6^ M, 9.09 $\times$ 10^−6^ M, 13.0 $\times$ 10^−6^ M, 20.0 $\times$ 10^−6^ M, 25.9 $\times$ 10^−6^ M, 31.0 $\times$ 10^−6^ M and 33.3 $\times$ 10^−6^ M for all (additions of 0μL, 100μL, 200μL, 300μL, 500μL, 700μL, 900μL, and 1000μL).

**Figure 4:**
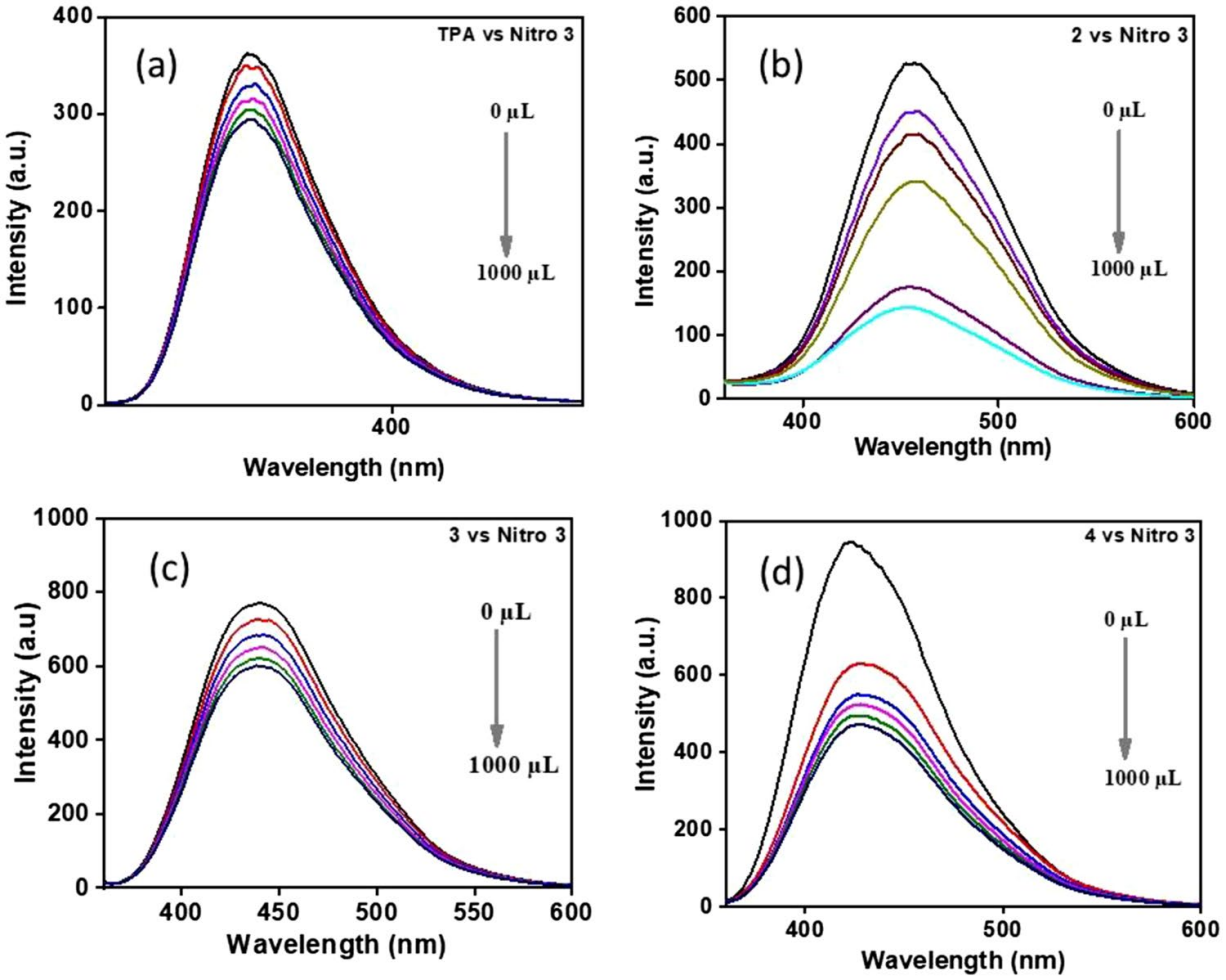
Fluorescence spectra of compounds **1**–**4** in the presence of 4-Nitrotoluene (Nitro 3). The concentration of 4-Nitrotoluene in DMSO is in the order of 0 M, 9.09 $\times$ 10^−5^ M, 16.6 $\times$ 10^–5^ M, 23.0 $\times$ 10^−5^ M, 28.5 $\times$ 10^−5^ M, and 33.3 $\times$ 10^−5^ M for all (additions of 0 μL, 200 μL, 400 μL, 600 μL, 800 μL, and 1000 μL).

To get an insight into the interaction of compounds, **1–4** with electron-deficient nitroaromatics such as 2,4,6-trinitrophenol (**PA**), 4-nitroaniline, 4-nitrotoluene, 4-nitrobenzoic acid, 4-nitrobenzaldehyde, and 2-nitrophenol absorption and emission studies were carried out. The studied NACs in our case caused quenching of fluorescence, out of which **PA** shows better quenching. In the case of **PA**, the colour change is observed for the naked eye. In compound **4,** the quenching was two-fold with PA might be due to the increased number of acid groups. In addition, this two-fold quenching may be due to excited state electron transfer between the twisted structure of the fluorophore and picrate anion. Similarly, the interaction of 4-nitrotoluene with **2** dramatically decreases the fluorescence intensity of the sensor, leading to an exciting result of a twofold quenching of fluorescence and high sensitivity of the sensor TPA di acid (**2**) towards the harmful nitrotoluene^36^. The results are shown in Figs. 3 and 4. All the other interactions are given in supplementary data. (Supplementary data)

**Figure 5:**
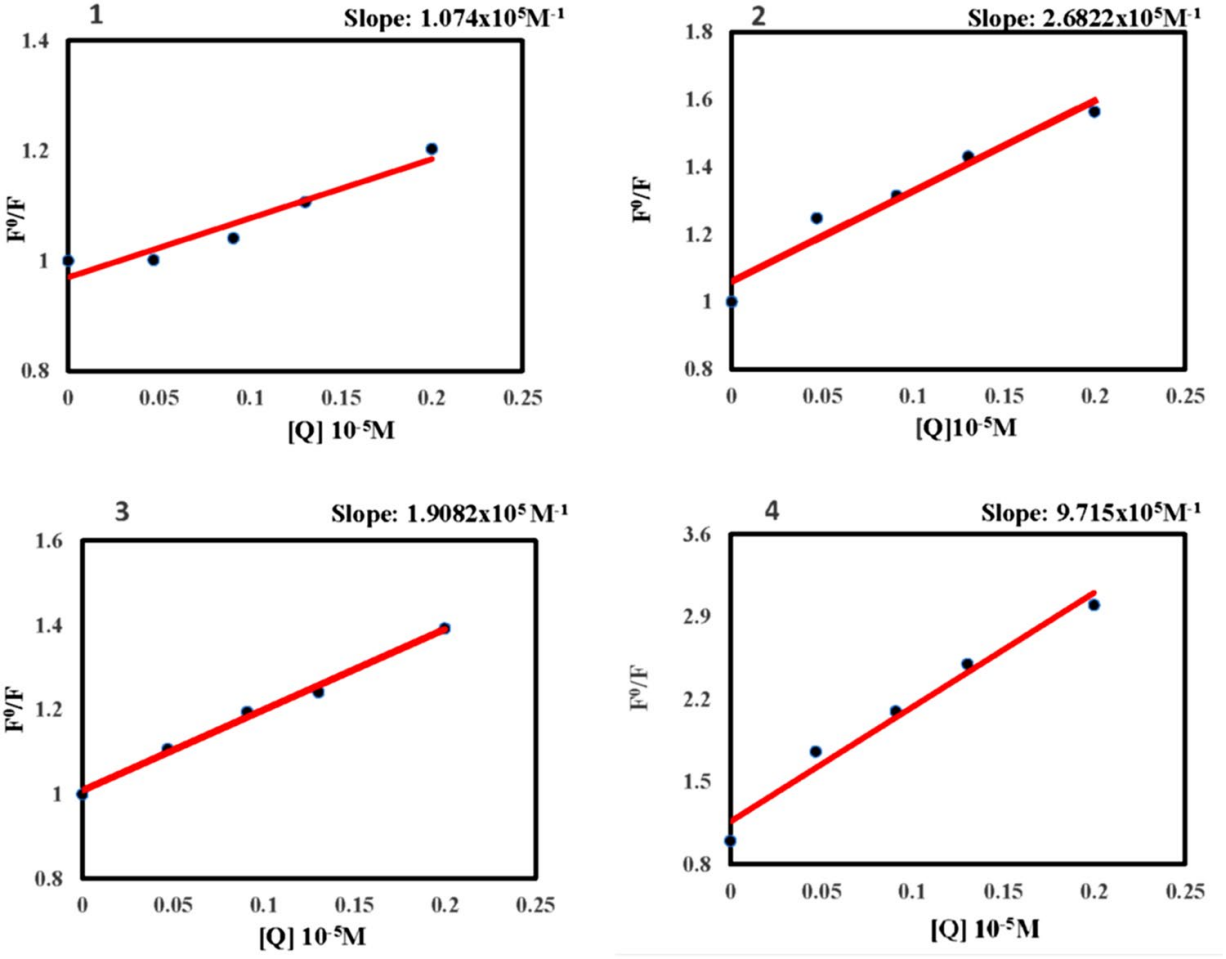
Stern–Volmer plots of compounds **1**–**4.**

The high quenching efficiency of PA can be attributed to deprotonation of strongly acidic phenolic –OH group followed by anion exchange with the sensor molecule^22^. In the case of conventional fluorophores^39^ featured with π- planar structures usually suffer from serious self-quenching in the aggregated state, poor photostability, and small Stokes’ shift values. In DMSO, PA undergoes deprotonation to form a picrate anion, which takes part in the ground state charge transfer complex formation. Thus, polar solvent like DMSO enhances the binding affinity of analyte and sensor. To gain an insight into the quenching efficiency, the Stern–Volmer quenching constant was calculated by the equation (I_0_/I = 1 + K_SV_ [Q]). Where I_0_ and I are the fluorescence intensity before and after the addition of PA, [Q] is the concentration of PA. The linearity of the plots supports the static quenching mechanism by a ground state charge transfer between the analytes and sensors^20^ (Fig. 5) Among the three compounds studied, **4** has shown better results with a high K_SV_ quenching constant value of 9.715 × 10^5^ M^−1^ due to the number of the acid groups increased indicating that the TPA tri acid molecule exhibited better binding interactions with the analyte. It also reveals that the TPA tri acid is the best candidate for working as a fluorescent sensor owing to its sensitivity^36,40^.

Whereas **1** and **3** showed relatively lower quenching constant values of 1.074 × 10^5^ M^1^ and 1.908 × 10^5^ M^−1^ respectively (Fig. 5) contributing to the idea of excited state twisted geometry leading to poor binding interactions. Micro molar level of detection was achieved with our molecules under study as pertinent with other fluorophores^38^ The reported molecules showed high Stokes’ shift as well as good quenching efficiency for nitroaromatics by overcoming the aforementioned issues such as self-quenching in the aggregated state and poor photostability.

### The response towards other NACs

Along with PA and 4-nitrotoluene, several other nitro compounds such as 4-ntroaniline, 4-nitrobenzoic acid, 4-nitrobenzaldehyde, and 2-nitrophenol were used to check the selectivity of the sensors. The quenching of the emission intensity in other NACs was found to be insignificant as compared to the cases with PA and 4-nitrotoluene.

### Solution mode detection

**Figure 6:**
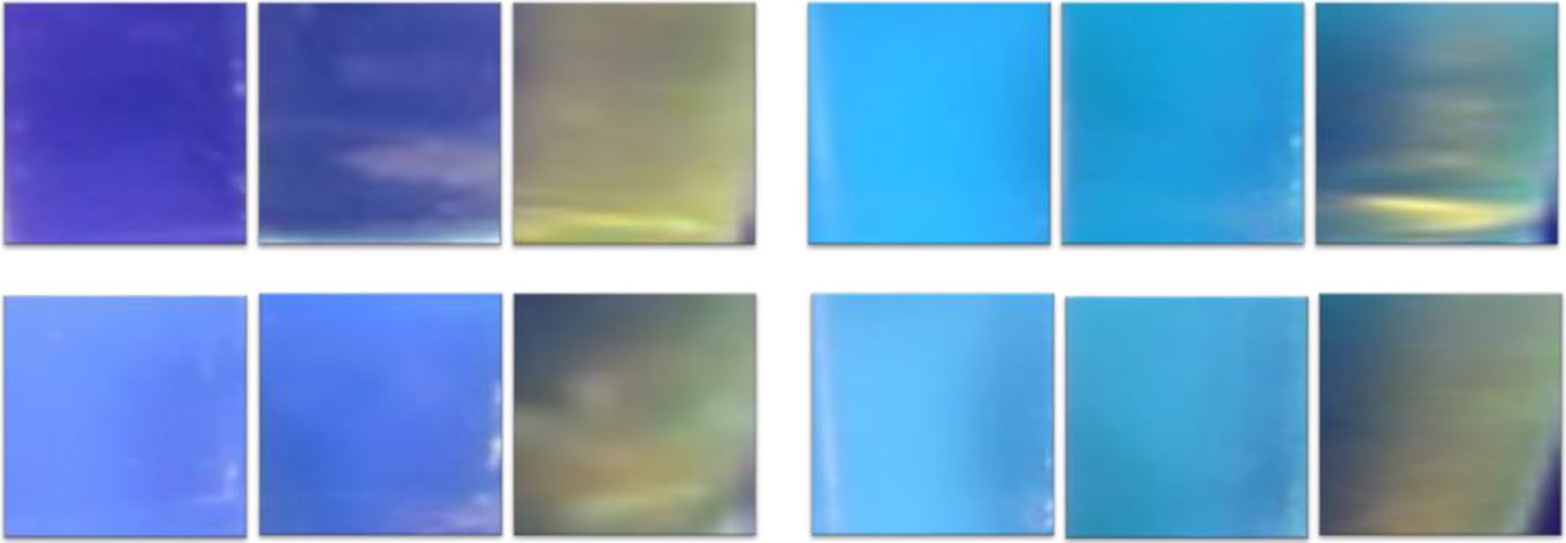
Visual color change (under UV light) upon gradual addition of 10^−4^ M PA to 4 mL of 10^−6^ M solution of compounds **1, 2, 3** and **4** in DMSO, (left, 0 μL; middle: 50 μL; right: 300 μL).

Visual detection of harmful nitroaromatic compounds is important for personal safety and environmental reasons, and low cost-high efficiency is an added benefit^39,41,42^. For visual detection, 10^−4^ M PA was slowly added to 10^−6^ M solutions of compounds **1, 2, 3,** and **4** incrementally as shown in Fig. 6. On subsequent addition of PA, the fluorescence quenched from blue to non-fluorescent, which is consistent with the previously obtained result^26^.

### Contact mode detection

**Figure 7:**
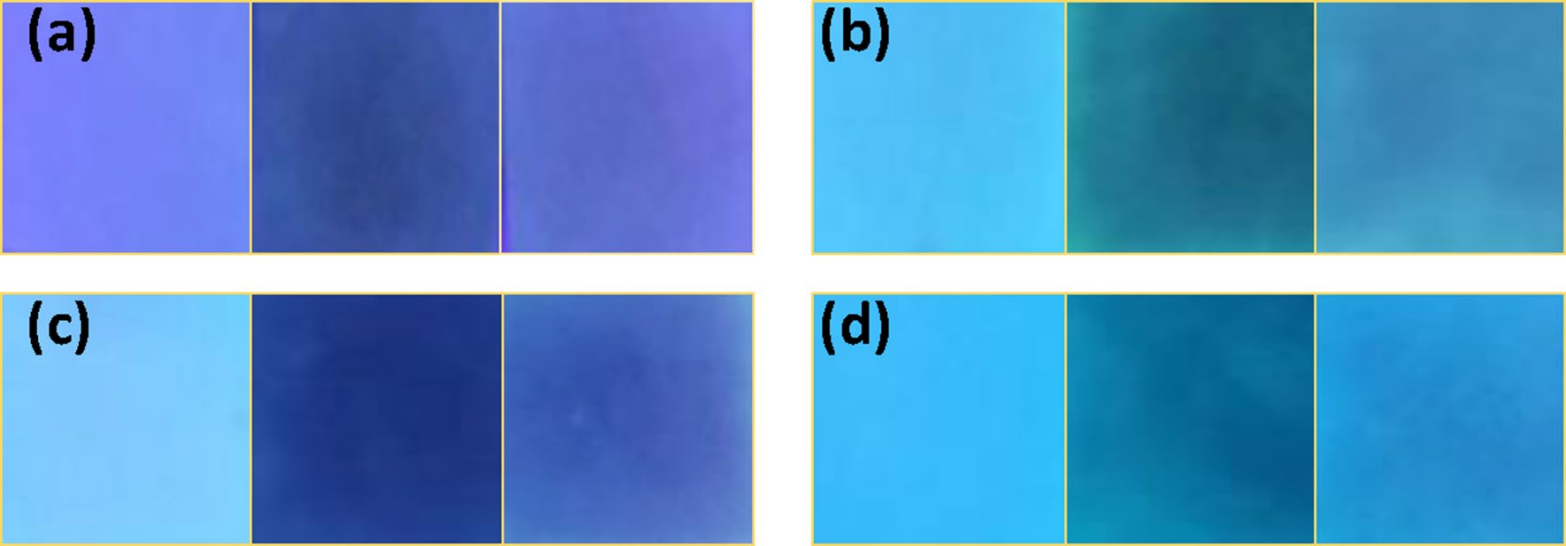
Paper strip images of compounds **1**–**4** after the addition of different concentrations of PA. (0 M, 10^−3^ M, 10^−11^ M).

For contact-mode detection, Whatman 42 filter paper was cut into 2 cm^2^ pieces and dipped into the concentrated solutions of the compounds **1–4** for 10 min and dried subsequently under reduced pressure. After complete drying, the strips were introduced to different concentration solutions of PA (from 10^−3^ to 10^−11^ M). 10μL of PA was drop-casted onto the freshly prepared test strips. The test strips were then analyzed under a UV lamp and given in Fig. 7. Dark spots were obtained for concentrated PA. The colour of the spot faded upon decreasing the concentration.

### Computational studies

**Figure 8:**
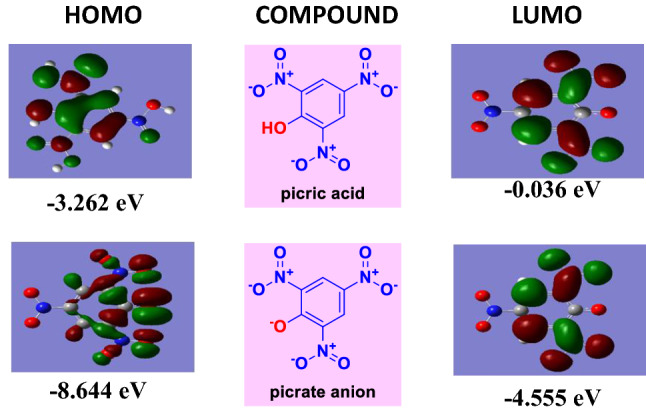
HOMO–LUMO of picric acid and picrate anion.

**Figure 9:**
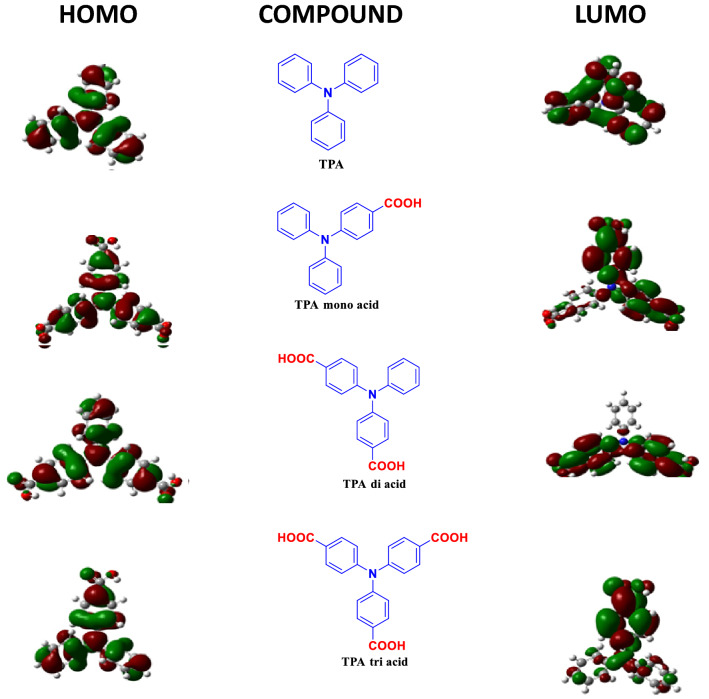
HOMO–LUMO of compounds **1**–**4.**

To get an idea about the electronic distribution in the frontier orbitals of compounds, density functional theory (DFT) calculations were carried out. The molecules were optimized at the 6–31 D basis level of DFT using Gaussian and shown in Fig. 8. Frontier molecular orbital values (HOMO and LUMO) are given in Fig. 9 and the HOMO–LUMO energy levels of Compounds **1**–**4** are given in Table 2. The fluorescence quenching mechanism can be explained by donor–acceptor charge transfer between picrate anion and sensor molecule. Charge transfer is the probable way of quenching fluorescence as the HOMO of compounds 1–4 is situated near the LUMO of the picrate. The LUMO of picrate anion (4.5 eV) is situated near the HOMO of fluorophores (5.7 eV). The feasible mechanism of fluorescence quenching stipulates the idea of a ground state charge transfer from sensor molecules to the electron-deficient PA which was also evident from the DFT calculation^22^.

**Table 2:** Listing the HOMO–LUMO energy levels of Compounds **1**–**4.**

Compound	Molecular formula	DFT energy (Hartree)	HOMO (eV)	LUMO (eV)	Band gap (eV)	Dipole D
1	C_18_H_15_IN	− 749.524	− 5.2420	− 0.8172	4.42487	0.0067
2	C_19_H_15_NO_2_	− 938.020	− 5.7142	− 1.6833	4.03085	6.8238
3	C_20_H_15_NO_4_	− 1126.51	− 6.1057	− 2.1913	3.91438	7.1509
4	C_21_H_15_NO_6_	− 1315.43	− 6.4434	− 2.4504	3.99303	2.9945

## Conclusion

In summary, we have developed a set of carboxylic acid group decorated triphenylamines (**2–4**) for the efficient detection of nitroaromatics. The molecules showed appreciable quenching in fluorescence by nitroaromatics. Our molecules showed high Stokes’ shift (126.8 nm for compound **2**) as well as good quenching efficiency. From photophysical studies, it is evident that compounds **2–4** underwent concentration-dependent fluorescence quenching by π-π interaction. Solution state fluorescence titration study revealed that all of the compounds have a high binding affinity (quenching constant value of compound **4** is 9.715 × 10^5^ M^−1^) for picrate anion. The quenching of fluorescence may be due to the sensor to picrate charge transfer in ground-state complex formation as well as resonance energy transfer between picrate and sensor molecules. DFT calculations gave an insight into the energy levels of the molecules and PA. From the contact mode, it was concluded that these acids can be used as dip-strip sensors for picric acid cost-effectively.

## Supplementary Information

Supplementary Information.
