# Supplementary material for: Fluorescence quenching based detection of nitroaromatics using luminescent triphenylamine carboxylic acids
Source: Sci Rep. 2021 Sep 29;11:19324. doi: 10.1038/s41598-021-97832-0 (PMC8481287; doi:10.1038/s41598-021-97832-0)
Supplement: Supplementary file 1 — Supplementary Information. [file 41598_2021_97832_MOESM1_ESM.docx]

**Supporting Information**

**for**

Fluorescence Quenching based detection of nitroaromatics using luminescent triphenylamine carboxylic acids

Aamnayee Mishra,^1^ R. Dheepika,^1^ P. A. Parvathy,^1^ P.M. Imran,^2^

N. S. P. Bhuvanesh,^3^ S. Nagarajan^1^*

**Materials and methods**

All the chemicals used were the highest grade commonly available and used as received. All solvents used were AR grade and were used without further purification. UV-Vis absorption spectra were recorded using a Jasco V-670 spectrophotometer. Perkin Elmer LS 55 spectrofluorimeter was used to obtain the emission spectra. NMR spectra were recorded using Bruker 400 MHz instrument in CDCl_3_ and d_6_-DMSO. The electrospray ionization mass (ESI-MS) spectra were recorded using THERMO Scientific Exactiveplus UHPLC MS spectrometer. Microwave-assisted synthesis was performed using a CEM microwave synthesizer. Column chromatography was carried out with slurry-packed activated silica gel (100-200 mesh). All the reactions and chromatographic separations were monitored by commercial TLC plates. For DFT studies, the molecules were optimized at the 6-31 D+ basis level of DFT using Gaussian. . Single-crystal x-ray diffraction studies data were collected on a BRUKER APEX2 X˗ray (three˗circle) diffractometer with Mo˗kɑ λ = 0.70173 Ǻ radiation. Single-crystal XRD was recorded on a BRUKER APEX2 X˗ray (three˗circle) diffractometer. The single crystal was obtained by slow evaporation of ethanol solution. Crystal was monoclinic with space group P 1 21/n (Figure 1). The crystal refinement parameters are given in Table 1. Crystal was obtained by slow A Leica MZ 75 microscope was used to identify a suitable colorless plate with very well defined faces with dimensions (max, intermediate, and min) 0.42 x 0.373 x 0.344 mm3 from a representative sample of crystals of the same habit.

**Table S1. Crystal data and structure refinement for compound 2**

**Data Compound 2**

Empirical formula C_19_H_15_NO_2_

Formula weight 289.32

Temperature 110.0 K

Wavelength 0.71073 Å

Crystal system Monoclinic

Space group P 1 21/n 1

Unit cell dimensions a = 9.1921(6) Å, α= 90°.

b= .6185(7) Å, β= 90.068(2)°.

c = 17.1694 (11) Å, γ= 90°.

Volume 1518.02(18) Å3

Z 4

Density (calculated) 1.266 Mg/m3

Absorption coefficient 0.082 mm-1

F(000) 608

Crystal size 0.241 x 0.172 x 0.045 mm3

Theta range for data collection 2.372 to 25.403°.

Index ranges -11<=h<=11, -11<=k<=11, -20<=l<=20

Reflections collected 15772

Independent reflections 2779 [R(int) = 0.0766]

Completeness to theta 25.242° 99.9 %

Absorption correction Semi-empirical from equivalents

Max. and min. transmission 0.4892 and 0.3109

Refinement method Full-matrix least-squares on F2

Data / restraints / parameters 2779 / 0 / 200

Goodness-of-fit on F2 1.112

Final R indices [I>2sigma(I)] R1 = 0.0651, wR2 = 0.1676

R indices (all data) R1 = 0.0811, wR2 = 0.1810

Extinction coefficient 0.033(4)

Largest diff. peak and hole 0.314 and -0.363 e.Å-3

**Figure S1**. ^1^H NMR spectrum of TPA monoacid (compound **2**)


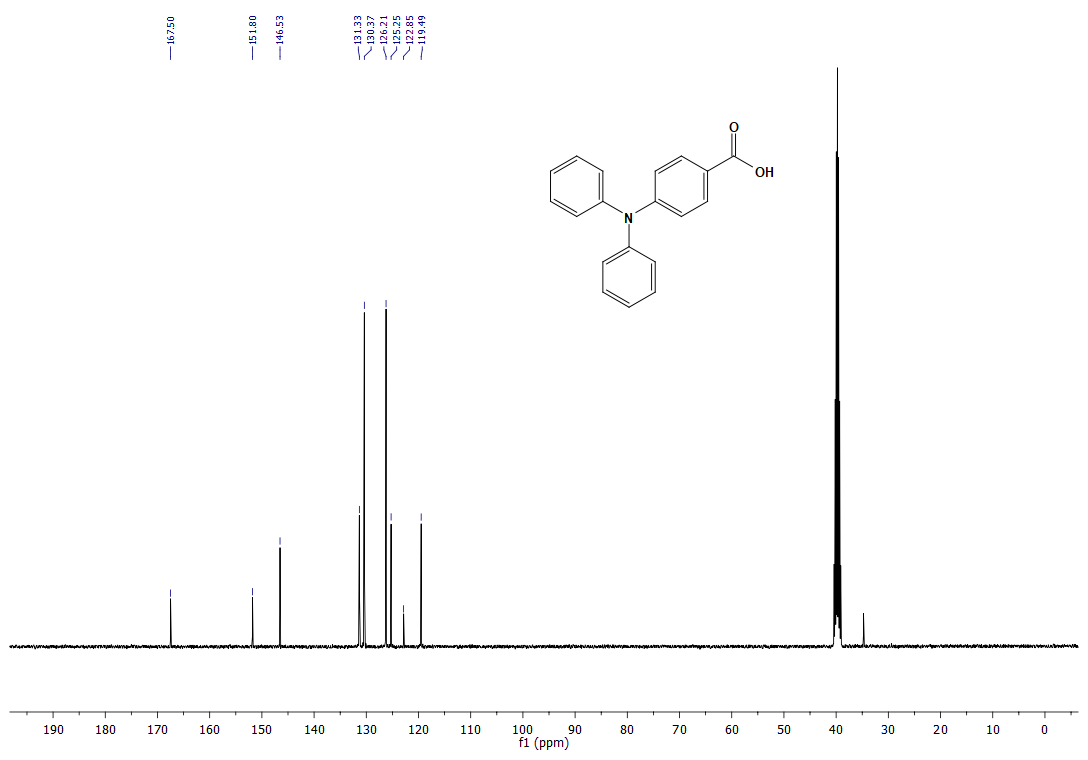


**Figure S2**. ^13^C NMR spectrum of TPA monoacid (compound **2**)

**
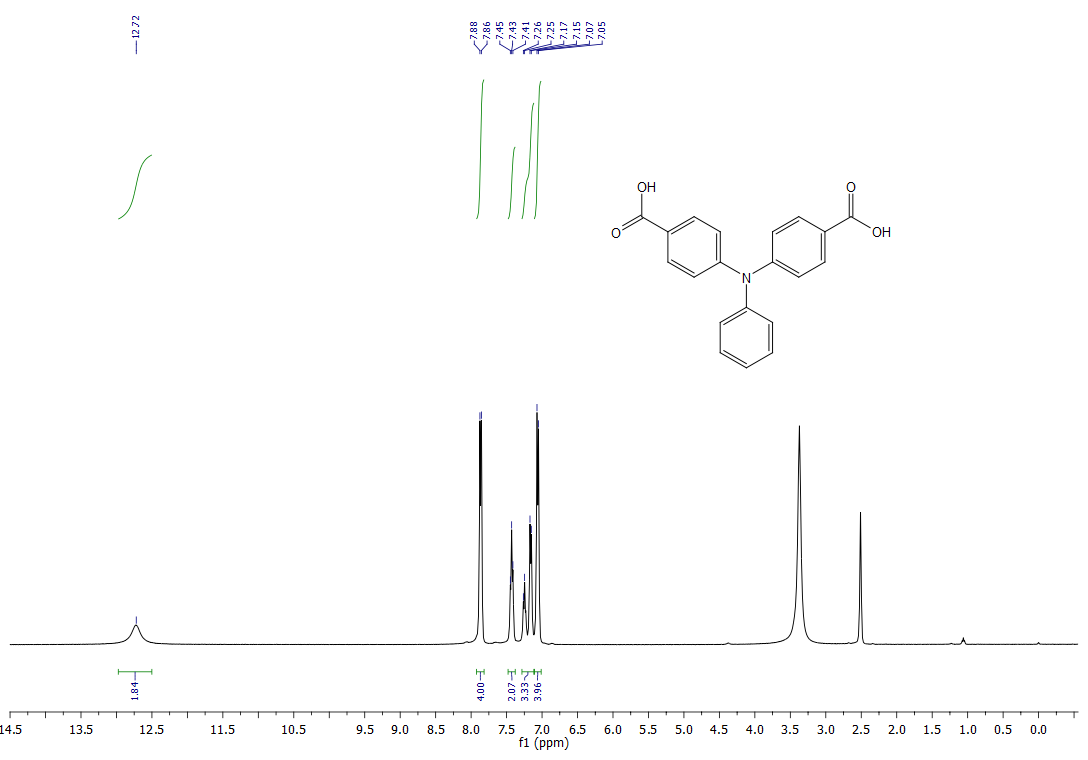
**

**Figure S3.**^1^H NMR spectrum of TPA di acid (compound **3**)**
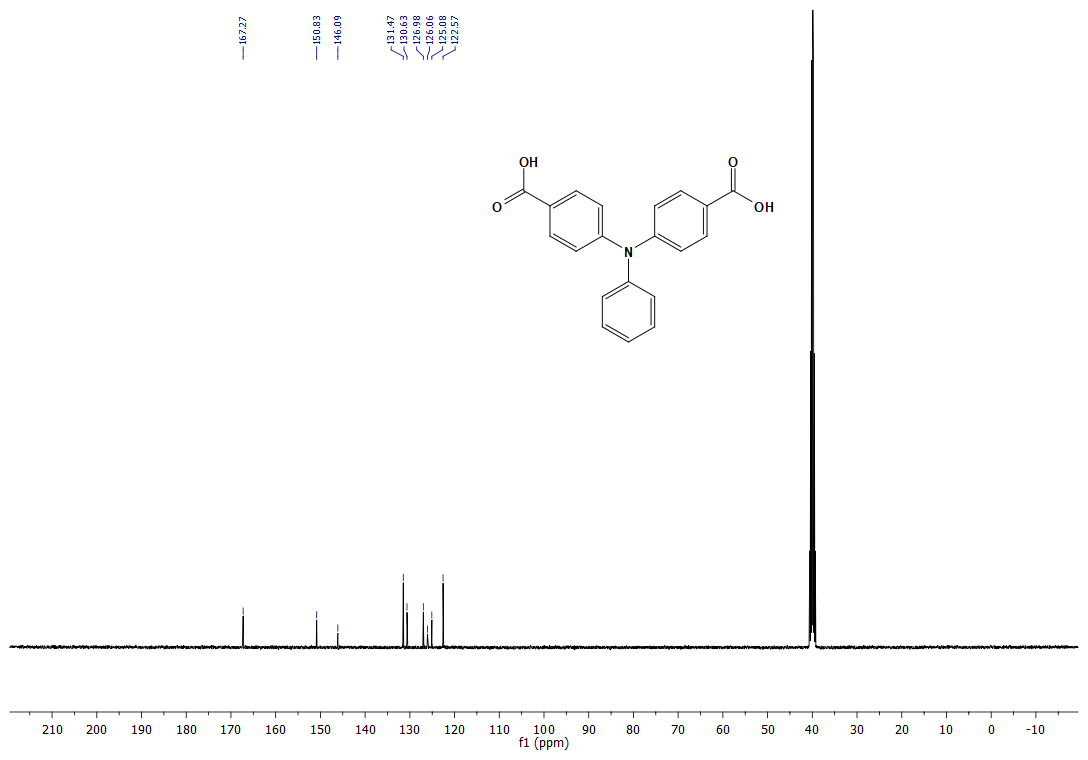
**

**Figure S4.** ^13^C NMR spectrum of TPA di acid (compound **3**)


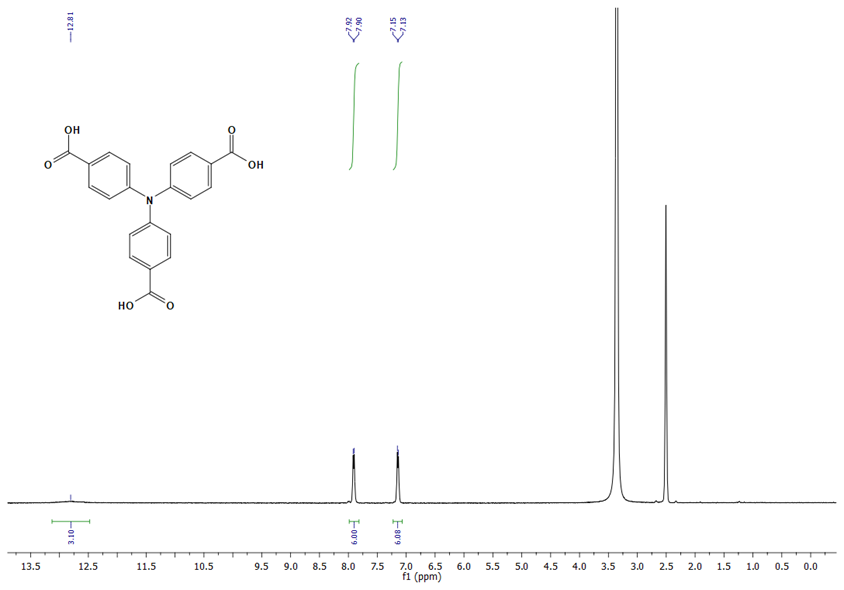


**Figure S5**. ^1^H NMR spectrum of TPA tri acid (compound **4**)

**
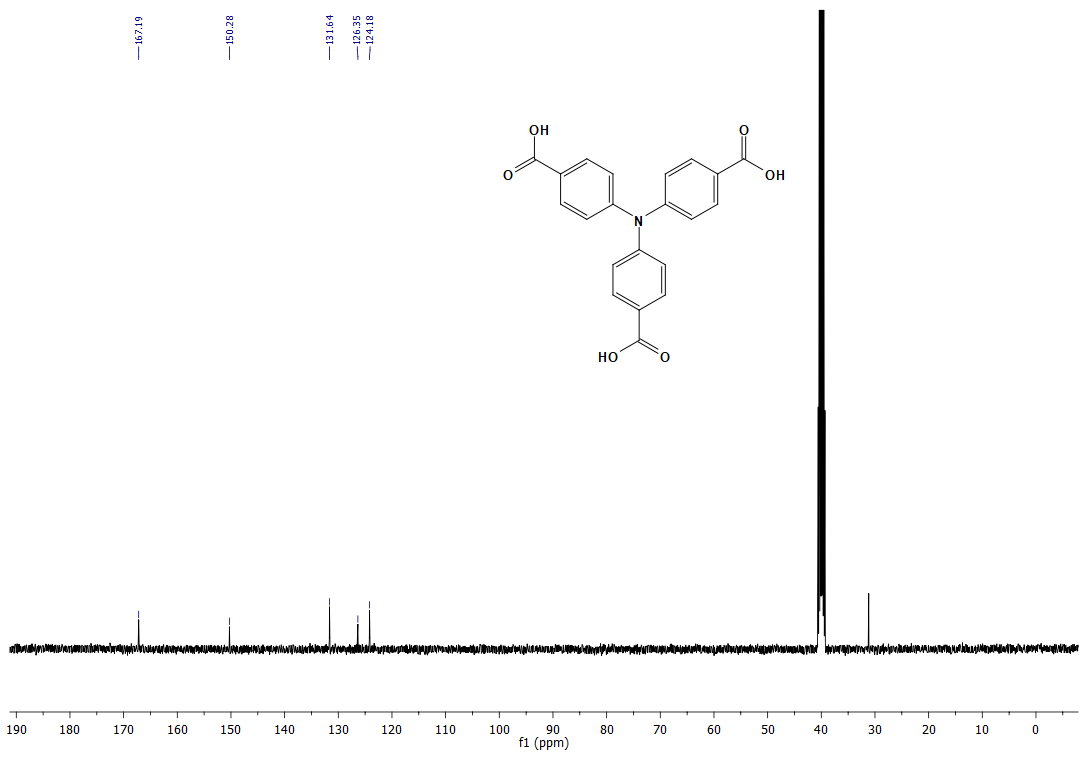
**

**Figure S6.** ^13^C NMR spectrum of TPA tri acid (compound **4**)

**Figure S7.** HRMS spectrum of TPA monoacid (compound **2**)

**Figure S8**. HRMS spectrum of TPA di acid (compound **3**)

**Figure S9**. HRMS spectrum of TPA tri acid (compound **4**)

**Table S2. Nitroaromatic compounds used in the studies**

| S. No. |  | NACs | Molecular weight (g/mol) | Named as |
| --- | --- | --- | --- | --- |
| 1  2  3  4  5  6 |  | 2,4,6-Trinitrophenol  4-Nitroaniline  4-Nitrotoluene  4-Nitrobenzoic acid  4-Nitrobenzaldehyde  2-Nitrophenol | 229.103  138.124  137.136  167.118  151.119  139.108 | PA  Nitro-2  Nitro-3  Nitro-4  Nitro-5  Nitro-6 |

**Interaction of nitroaromatics with the compounds 1-4**

**Figure S10**. (a) Absorption spectra of 2,4,6-Trinitrophenol (PA) with compounds **1**-**4**

**Figure S11**. (a) Absorption and (b) Emission spectra of 4-Nitroaniline (Nitro-2) with compounds **1**-**4**

**Figure S12**. (a) Absorption and (b) Emission spectra of 4-Nitrotoluene (Nitro-3) with compounds **1**-**4**

**Figure S13**. (a) Absorption and (b) Emission spectra of 4-Nitrobenzoic acid (Nitro-4) with compounds **1**-**4**

**Figure S14**. (a) Absorption and (b) Emission spectra of 4-Nitrobenzaldehyde (Nitro-5) compounds **1**-**4**

**Figure S15**. (a) Absorption and (b) Emission spectra of 2-Nitrophenol (Nitro- 6) with compounds **1**-**4**

**Figure S16.** Optimized structures of compounds **1-4**
